# Phenotypic and functional stability of leukocytes from human peripheral blood samples: considerations for the design of immunological studies

**DOI:** 10.1186/s12865-019-0286-z

**Published:** 2019-01-18

**Authors:** Adriana Navas, Lina Giraldo-Parra, Miguel Darío Prieto, Juliana Cabrera, María Adelaida Gómez

**Affiliations:** 1 Centro Internacional de Entrenamiento e Investigaciones Médicas-CIDEIM, Cali, Colombia; 2 Universidad Icesi, Cali, Colombia

**Keywords:** PBMC, Stability, PCR, ELISA, Leukocytes

## Abstract

**Background:**

Human peripheral blood mononuclear cells (PBMCs) are extensively used for research of immune cell functions, identification of biomarkers and development of diagnostics and therapeutics for human diseases, among others. The assumption that “old blood samples” are not appropriate for isolation of PBMCs for functional assays has been a dogma in the scientific community. However, partial data on the impact of time after phlebotomy on the quality and stability of human PBMCs preparations impairs the design of studies in which time-controlled blood sampling is challenging such as field studies involving multiple sampling centers/sites. In this study, we evaluated the effect of time after phlebotomy over a 24 h time course, on the stability of human blood leukocytes used for immunological analyses. Blood samples from eight healthy adult volunteers were obtained and divided into four aliquots, each of which was left in gentle agitation at room temperature (24 °C) for 2 h (control), 7 h, 12 h and 24 h post phlebotomy. All samples at each time point were independently processed for quantification of mononuclear cell subpopulations, cellular viability, gene expression and cytokine secretion.

**Results:**

A 24 h time delay in blood sample processing did not affect the viability of PBMCs. However, a significantly lower frequency of CD3+ T cells (*p* < 0.05) and increased LPS-induced CXCL10 secretion were observed at 12 h post-phlebotomy. Alterations in *TNFα, CCL8, CCR2* and *CXCL10* gene expression were found as early as 7 h after blood sample procurement.

**Conclusions:**

These data reveal previously unrecognized early time-points for sample processing control, and provide an assay-specific time reference for the design of studies that involve immunological analyses of human blood samples.

**Electronic supplementary material:**

The online version of this article (10.1186/s12865-019-0286-z) contains supplementary material, which is available to authorized users.

## Background

Human peripheral blood mononuclear cell (PBMCs) preparations, consisting primarily of lymphocytes (T cells, B cells and NK cells) and monocytes, are one of the most widely used samples in biomedical research. PBMCs are employed in a wide array of assays that range in complexity from simple cytotoxicity evaluations to sophisticated single cell functional and phenotypic immunological or molecular assays [1–3]. In vitro, phenotypic characterization and functional evaluation of human cells is one of the pillars for the development of clinically implementable diagnostic, prognostic and therapeutic tools [4–9]. Therefore, standardization and reproducibility of these assays is critical for their routine performance and the reliability of results.

PBMCs are obtained mainly by density gradient centrifugation, a technique described by Boyum in 1968 [10]. Several factors could influence the quality of PBMCs preparations, such as the conditions of sample collection [11] (type of anticoagulant used and blood collection rate), sample processing time [12], technique used to obtain the PBMCs (apheresis vs. density gradient centrifugation) [13], and finally their preservation [14, 15]. These factors influence cell viability and cell count, gene and protein expression (e.g. surface markers), responses to mitogenic stimuli [16], impact cell functions and ultimately create bias in the study results.

Clinical studies that involve the use of human blood samples for analysis of immunological cell functions require strategies to optimize and standardize pre-analytical processes to minimize the variability of the data, maximize reproducibility of the assays and ultimately assure quality of the generated data. These strategies involve standardization of 1) the sampling technique: defining the time of day for sample procurement, the use of, and type of anticoagulant, the blood volume to be obtained, etc.; 2) sample handling: defining whether blood samples will be maintained in agitation or resting and at what temperature prior to analysis; 3) transportation: establishing the method of transportation to be used throughout the study, and the time from sample procurement to sample processing; 4) sample processing: methods for cell isolation and purification; and 5) storage: defining the medium and long term storage temperature, the minimum and maximum storage time, storage medium, among others. Establishing these parameters is particularly challenging for multicentric studies, where samples need to be collected at different clinical sites, introducing variability in some aspects such as the time between sample procurement and processing.

Although there is a general assumption in the community that “old blood samples” (without a precise time definition for “old”) should not be used, there is no systematic data on the impact of time after phlebotomy on the stability of PBMCs from human blood samples. In this study, we aimed to systematically assess the effect of time, over a 24 h time frame after phlebotomy, on the stability of human PBMCs used for downstream immunological analyses.

## Methods

### Study design

**Fig. 1:**
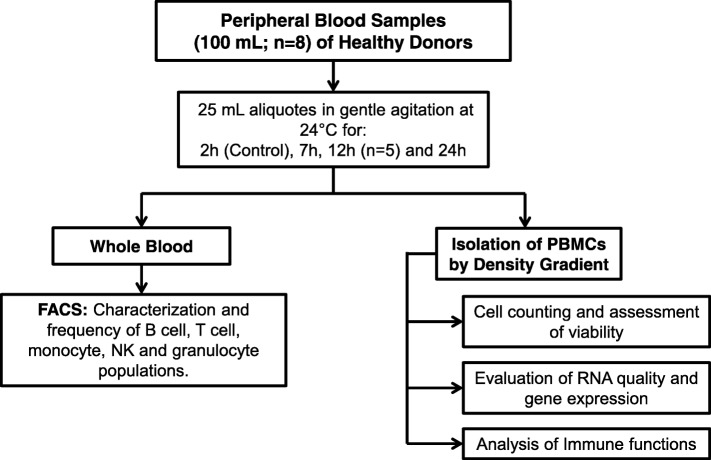
Schematic Workflow. Peripheral blood samples (100 mL) from 8 healthy donors were obtained by venipuncture and divided into four aliquots and left in gentle agitation at room temperature (24 °C) for 2 h (control), 7 h, 12 h (*n* = 5) and 24 h post phlebotomy. Cellular viability and counting, expression of immune-related genes, and functional evaluations were performed at each time point

We evaluated the stability of human mononuclear cell populations in peripheral blood samples from eight healthy volunteers over a 24 h time frame after phlebotomy. Eligibility criteria included age between 18 and 60 years, no co-morbidities, no immunosuppressive conditions, no pregnancy or lactation and a negative human immunodeficiency virus (HIV) test. Blood samples (100 mL) were obtained by venipuncture of an arm vein, collected on 50 mL polypropylene tubes (Falcon, #352098), and anticoagulated with EDTA. Each sample was divided into four aliquots (25 mL) and left in gentle agitation using a Vari-Mix tube rocker at room temperature (24 °C) for 2 h (control), 7 h, 12 h and 24 h post phlebotomy (Fig. 1). Each 25 mL blood aliquote was independently processed for quantification of leukocyte subpopulations, isolation of mononuclear cells for assessment of viability, gene expression and functional analyses (in vitro cytokine production).

### Characterization of cell populations by fluorescence-activated cell sorting (FACS)

One milliliter of blood from each time point (2 h, 7 h, 12 h and 24 h post phlebotomy) was used to quantify the relative frequencies of cell lineages (T and B lymphocytes, NK cells and monocytes) and the expression intensity of lineage markers (determined by the mean fluorescence intensity, MFI). This was achieved using the BD human Monocyte/NK cell (CD14/CD19/CD16/CD56) antibody cocktail (BD Biosciences, catalog #562,089) and PE-Cy™7 Mouse Anti-Human CD3-Clone SK7 (BD Biosciences, catalog # 557851), according to the manufacturer’s instructions. Flow cytometry acquisition was performed on a BD Accuri C6 (BD Biosciences) cytometer; 50,000 events were collected for each processed sample. To ensure the quality of measurement, the performance of the cytometer was evaluated using validation beads to ensure coefficients of variation < 5% for the top peaks on each fluorescence detector and the forward scatter. Furthermore, fluorescence compensation was conducted before each sample acquisition. Data analysis was done using Flow-Jo (Treestar) version 10.0. The gating strategy included a selection of monocytes and B cells based on CD14+ and CD19+ expression and FSC/SSC (forward and side scatter features). NK cells and T cells were defined on total lymphocytes population by FSC/SSC and the expression of CD16 + CD56+ (NK) and CD3+ (T cells). The granulocyte population was defined based on FSC/SSC.

### Peripheral blood mononuclear cells isolation

PBMCs were obtained by centrifugation of PBS-diluted (1:1) blood samples over a Ficoll-Hypaque gradient (Histopaque-1077, Sigma-Aldrich) following the manufacturer’s instructions. Cell viability was evaluated by exclusion of Trypan blue dye (GE Healthcare Life Sciences) at a 1:10 dilution and estimated as absolute numbers of viable and non-viable cells and as percent cell viability. Ten million PBMCs were collected, centrifuged at 450 x *g* and the pellet was resuspended in 1 mL TRIzol ® (Invitrogen, USA), stored at − 80 °C for 72 h and used for RNA extraction to evaluate gene expression. The remaining cells were stored at − 80 °C for one week in fetal bovine serum (FBS) complemented with 10% dimethyl sulfoxide (DMSO). Subsequently, cells were gently thawed on a 37 °C water bath, washed twice with RPMI containing 10% FBS and immediately processed for evaluation of cell functions as described below.

### Real-time PCR and RNA extraction

Total RNA was extracted from samples stored in TRIzol ®, as described by the manufacturer. The quantity and quality of the extracted RNA was evaluated in a Nanodrop ND-1000 spectrophotometer and the RNA integrity was evaluated by electrophoresis in 1.3% agarose gels. cDNA was synthesized using a high-capacity cDNA reverse transcription kit (Thermofisher Scientific). Gene expression of inflammatory mediators was evaluated using Taqman probes (Thermofisher Scientific) for *CCL8* (Hs00271615_m1), *CCL2* (Hs00234140_m1), *CXCL3* (Hs00171061_m1), *CXCL10* (Hs01124251_g1), *CCR2* (Hs00704702_s1), *CCR7* (Hs01013469_m1), *TNF*α (Hs00174128_m1), *IL-1ß* (Hs01555410_m1) and *GAPDH* (HS99999905_m1), in a BioRad® CFX-96 detection platform. Gene expression was quantified by the ΔΔCt method using GAPDH as the normalizing gene, and contrasting against control cells (2 h post-phlebotomy). Gene expression was expressed as fold change (2^-ΔΔCt^).

### Secretion of TNF-α, IL-1β and CXCL10

PBMCs were seeded in 12 well plates at 5 × 10^6^ cells/well in 1 mL of RPMI supplemented with 10% FBS. Cells were stimulated with 1 μg/mL ultrapure Lipopolysaccharide (LPS) (Sigma-Aldrich) for 12 h or left untreated. Culture supernatants were collected, and the secretion of TNFα, IL-1β and CXCL10 was assessed by enzyme-linked immunosorbent assay (ELISA) using R&D system Human TNFα, IL-1β and CXCL10 DuoSet ELISA kits according to the manufacturer’s recommendations. Absorbance readings at 450 nm were performed on a Dinex microplate reader.

### Statistical analysis

The Kolmogorov-Smirnov test was used to determine the parametric or nonparametric distribution of the data. One-way analysis of variance (ANOVA) or Kruskal-Wallis test followed by multiple-comparison tests were employed for group comparisons. Statistical significance was defined at a *p* value < 0.05. All data were analyzed using Prism 6 software.

## Results

### The frequency of monocytes, NK cells, B cells and granulocytes, but not T cells, is maintained up to 24 h post-phlebotomy

**Fig. 2:**
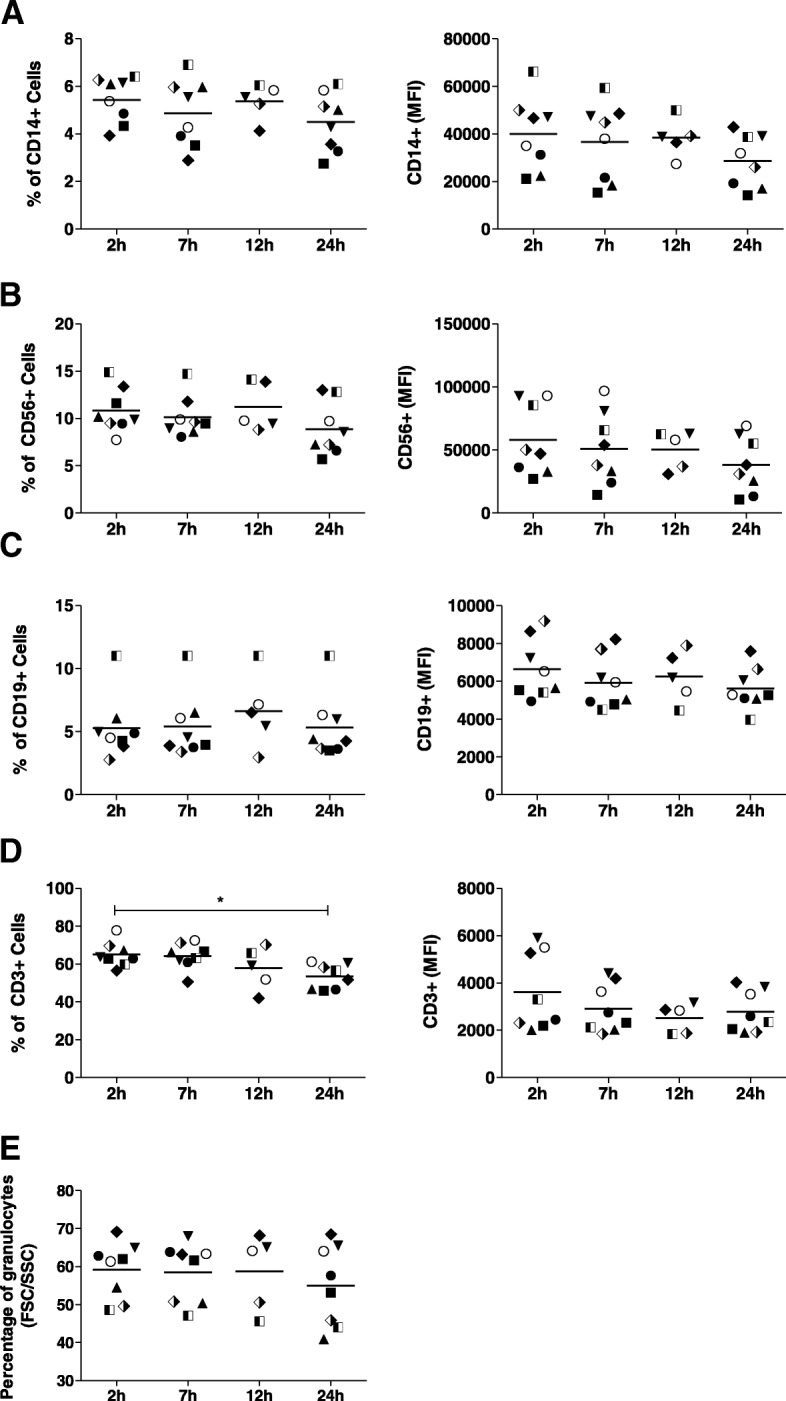
Phenotypic characterization of immune cell populations in peripheral blood. Frequencies (left column) and mean fluorescence intensity (right column) of CD3+, CD14+, CD56+ and CD19+ cells (**a**-**d**). Granulocytes were defined by forward and side scatter properties (**e**). Data are presented as mean or median depending on the distribution of the data. Statistical significance was estimated by one-way ANOVA or Kruskal-Wallis. *P < 0.05 compared to the control (2 h)

Analysis of cell surface marker expression was conducted to evaluate the impact of time after phlebotomy on specific immune cell populations. The frequency of monocytes, NK cells, B cells (determined by expression and mean fluorescence intensity –MFI- of CD14, CD56 and CD19, respectively,) and granulocytes, was unaltered up to 24 h post phlebotomy (Fig. 2a-e). The frequency of CD3+ T cells significantly decreased from an average 65.06% at 2 h to 53.46% at 24 h (*p* < 0.05), despite no significant difference in the CD3 MFI (Fig. 2d). Decrease of the CD3+ cell population was accompanied by increase in CD3- lymphocytes (Additional file 1: Figure S1), suggesting time-dependent changes in expression of CD3, rather than an impact on cellular viability.

### Viability of PBMCs and cell counts are maintained up to 24 h post-phlebotomy

**Fig. 3:**
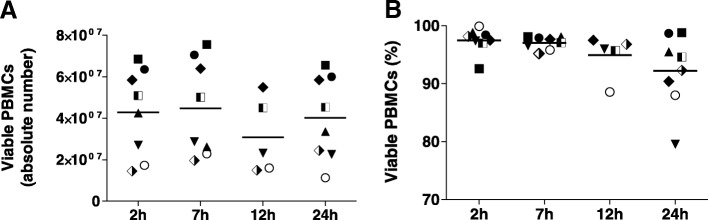
Evaluation of cell counts and viability. Absolute counts of viable PBMCs (**a**) and their frequency (**b**) are presented as scatter dot plots and median. Statistical significance was estimated by one way Kruskal-Wallis

PBMCs were isolated from peripheral blood and cell viability was measured. Absolute counts of viable cells were similar in all samples and across all time points (Fig. 3a). Likewise, the frequency of viable PBMCs was above 90% in samples processed at 2 h, 7 h and 12 h from all donors. A slight but non-significant reduction in the frequency of viable cells was observed at 24 h (Fig. 3b); however, cell viability was maintained above 85% for all except for one sample. Concordant with viability assays of isolated PBMCs, no significant difference in cell viability was observed for total leukocyte preparations (2 h: 97.06% ± 1.88 and 24 h: 94.9% ± 0.17).

### Expression of immune related genes in PBMCs is altered by the time to sample processing

**Fig. 4:**
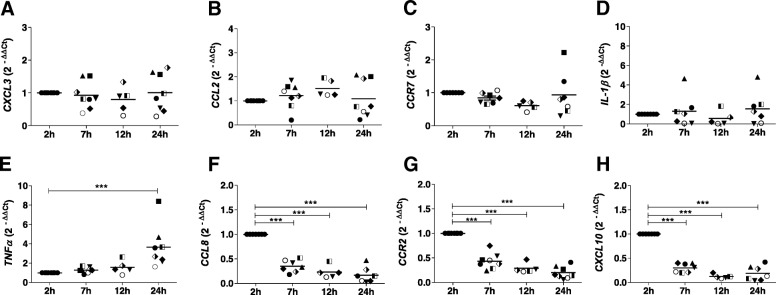
Chemokine and cytokine gene expression in PBMCs. Gene expression of *CXCL3*, *CCL2*, *CCR7*, *IL-1ß*, *TNFα*, *CCL8*, *CCR2* and *CXCL10* (**a**-**h**), was measured by qRT-PCR. Data are presented as mean and statistical significance estimated by one-way ANOVA followed by Dunnett’s multiple comparison test. *: *p* < 0.05, **: *p* < 0.01, ***: *p* < 0.001

Gene expression of eight inflammatory mediators involved in the activation and recruitment of monocytes/macrophages (*TNFα, CCL8, CCL2, CCR2, IL-1*β), dendritic cells (*CCR7*), T cells (*CXCL10*) and neutrophils (*CXCL3*) was evaluated in isolated PBMCs in the absence of any ex-vivo stimulation. Expression of *CXCL3, CCL2, CCR7* and *IL-1*β was unaltered at all time points when compared to the 2 h control (Fig. 4a-d). However, expression of TNF*α, CCL8, CCR2* and *CXCL10* was affected by the time-to-sample processing. While *TNFα* gene expression was strongly and significantly induced (> 3 fold), the levels of *CCL8, CCR2* and *CXCL10* were repressed as early as 7 h after sample procurement (Fig. 4e-h). Quality assessment of the extracted RNA by Nanodrop profiles and visual check on agarose gels showed that the RNA integrity of all samples was preserved; indicating that modulation of gene expression was not a result of RNA degradation (Additional file 1: Table S1 and Additional file 1: Figure 2). No differences in *GAPDH* expression (normalizer gene) were observed among patients and between samples obtained at different time points (Additional file 1: Figure S3).

**Fig. 5:**
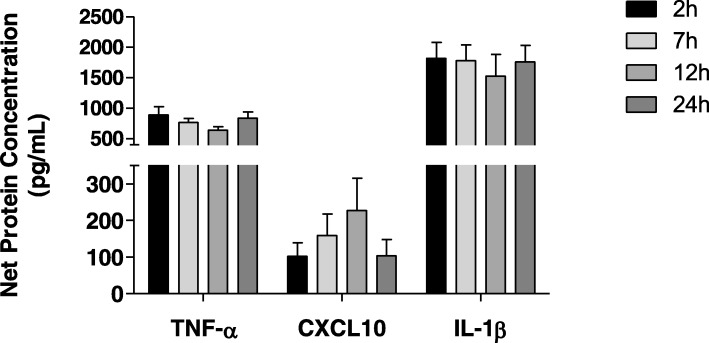
TNFα, IL1β and CXCL10 secretion in PBMCs. PBMCs were stimulated with 1 μg/mL ultrapure LPS (Sigma) for 12 h or left untreated. Cytokine secretion was measured in culture supernatants. Data are presented as mean ± SEM (*n* = 8) and statistical significance was estimated by one-way ANOVA

To assess whether the alterations in gene expression had a functional impact, secretion of TNFα, IL-1β and CXCL10 (each as a representative molecule of upregulated, unaltered and downregulated genes) was evaluated in LPS-stimulated PBMCs. Secretion of TNFα and IL-1β was homogeneous in all samples over the 24 h time frame (Fig. 5). CXCL10 secretion increased in a time-dependent manner up to 12 h and decreased at 24 h, however these changes were not statistically significant. These results suggest that despite a considerable effect elicited by the time-to-sample processing in the basal levels of gene expression, mitogen-induced protein secretion remains predominantly unaffected up to 24 h.

## Discussion

Harmonized and controlled experimental methods are critical for all clinical and non-clinical human studies. Routinely, the checkpoints for quality control of blood samples and its derivatives rest on variables such as sample processing procedures, anticoagulants used, temperatures, incubation/exposure times, brand and lot of reagents, among others. However, sample pre-processing parameters that could influence the quality and stability of the biological material such as time-to-sample processing or time of day for sample procurement are not often considered. Results from this study revealed that a time delay between obtaining a peripheral blood sample and its processing, affects inflammatory gene expression and potentially cell functions, providing critical evidence of the importance of controlling blood sample handling variables prior to ex-vivo analyses.

Previous studies have evaluated the impact of time after phlebotomy and its relationship with the quality of data derived from immunological analyses [12, 17–21]. Despite their important contribution, these studies evaluated the effect of sample processing delay in a single immunologic parameter (cell function, cell count, activation markers or gene expression), and usually during just one time point (most of them after a 24 h time frame), limiting the accurate definition of sample processing times, especially when studies involve multiple clinical sites and multiple immunological parameters. To overcome this, our approach aimed to concomitantly analyze the dynamic effects of time to blood sample processing on multiple immune and cellular parameters.

Although not statistically significant, our results showed a reduction in the cellular viability at 24 h, which could have important biological implications. Similar findings were previously reported, where average viability within 8 h of processing was ∼92%, decreasing significantly to ∼84% when processing was delayed 24 h, concluding that for optimal cell viability and recovery, PBMCs must be processed and cryopreserved within 8 h after venipuncture [18]. Our data extend these observations and indicate that in terms of cell viability, blood samples could be processed up to 12 h after phlebotomy.

In the context of immunological assays and analyses of cell function, the time frame between phlebotomy and PBMCs isolation is critical [15, 18]. A delay in the processing of PBMCs over 8 h, has been shown to result in alterations of NK cells functions: decreased expression of chemokine receptors (CCR4 and CCR7), reduction of degranulation capacity and secretion of IFNγ and TNFα [22]. Similarly, a progressive impairment of antigen presenting cell function of plasmacytoid and myeloid dendritic cells and monocytes has been reported at 0, 6, 12 and 24 h between venipuncture and PBMC isolation [23]. Our data showed that the frequency of monocytes, NK cells, and B cells in whole blood was unaffected at early time points and up to 24 h after phlebotomy, concurring with prior studies and expanding towards earlier time-points of analysis [24]. However, our results revealed a significant decrease in the CD3+ T cell population at 24. Loss of CD3+ T cells occurred concomitantly with increase in the CD3 negative lymphocyte population, suggesting antigen loss rather than an impact on cell viability. Rapid internalization and recycling of the TCR:CD3 complex has been reported in resting T cells [25], potentially explaining the observed reduction in the CD3+ T cell population.

In the context of immune-related gene expression, we observed different patterns of expression among the analyzed genes. Whereas expression of *TNFα* was strongly upregulated in a time-dependent manner, and *CCL8, CCR2* and *CXCL10* were down-regulated at all time points after 2 h, and the expression of *CXCL3, CCL2, IL-1β* and *CCR7* remained unchanged. Expression profiling in PBMCs using microarrays from human blood samples processed immediately or the next day after overnight incubation revealed that 31.7% of genes (2034 of the 6414 evaluated genes) were sensitive to ex-vivo incubation. A high proportion of these genes were involved in basic cellular processes such as transcriptional regulation, cell cycle progression, TNF-related functions, immune signaling, apoptosis and cytokines/chemokines signaling [12]. Interestingly, changes in gene expression have been reported to occur as early as 4 h post venipuncture [21]. These processes may reflect transcriptional events as an active response to cellular stress, and/or effects induced by alterations in cell-to-cell contact dynamics, which alert to an understanding of the basal gene expression patterns analyzed in blood samples prior to functional interpretation of gene expression profiles.

This study explored the effect of time to blood sample processing on a spectrum of immunological readouts. Overall, our findings indicated that blood samples processed after 12 h of phlebotomy should be carefully controlled and critically analyzed as alterations in cell viability and functionality are evidenced. This has important implications in multi-center/multi-site studies and field studies where time control can be challenging. Potential strategies to overcome this could include definition of a single time-point for sample processing within all clinical sites, inclusion of time-specific control samples to be collected simultaneously to the study samples (e.g. a blood sample from a study subject processed in parallel to one of a healthy volunteer as a normalizing control). Results presented herein provide an evidence base for the design of studies that involve human blood sampling and downstream analyses of immunological functions.

## Additional file

Additional file 1: **Figure S1.** Representative dot plot showing the effect of sample processing time in CD3+ expression in lymphocyte populations from whole blood. Lymphocyte population was gated based on FSC/SSC and the frequencies of expression for NK cell marker CD16 + CD56+ PE (y axis) and CD3 (x axis) were determine for each time point. **Table S1.** Evaluation of RNA in PBMCs samples. The quantity and quality of the extracted RNA was evaluated in the Nanodrop ND-1000 spectrophotometer taking into account the ratio of absorbance 260/280 and 260/230. **Figure S2.** Evaluation of RNA integrity in PBMCs samples. Agarose gel electrophoresis of RNA samples for confirmation of RNA integrity by inspection of the 28S and 18S rRNA bands. Control samples: Lanes 1, 2, 3, 10, 14, 18, 22 and 26; 7 h samples: Lanes 4, 5, 6, 11, 15, 19, 23 and 27; 12 h samples: Lanes 12, 16, 20, 24, and 28; 24 h samples: Lanes 7, 8, 9, 13, 17, 21, 25 and 29. **Figure S3.** Evaluation of *GAPDH* gene expression in PBMCs. Gene expression was measured by qRT-PCR, each figure represents the CT value and its stability over time for samples from each volunteer. Individual and mean values are shown. No statistical differences were observed as estimated by one-way ANOVA followed by Dunnett’s multiple comparison test. (DOCX 446 kb)

## Abbreviations

DMSO: Dimethyl sulfoxide
EDTA: Ethylenediaminetetraacetic acid
ELISA: Enzyme-linked immunosorbent assay
FACS: Fluorescence-activated cell sorting
FBS: Fetal bovine serum
HIV: Human immunodeficiency virus
LPS: Lipopolysaccharide
MFI: Mean fluorescence intensity
PBMCs: Peripheral blood mononuclear cells
RT-PCR: Real time polymerase chain reaction
