# Supplementary material for: Phenotypic and functional stability of leukocytes from human peripheral blood samples: considerations for the design of immunological studies
Source: BMC Immunol. 2019 Jan 18;20:5. doi: 10.1186/s12865-019-0286-z (PMC6339328; doi:10.1186/s12865-019-0286-z)
Supplement: Supplementary file 1 — Figure S1. Representative dot plot showing the effect of sample processing time in CD3+ expression in lymphocyte populations from whole blood. Lymphocyte population was gated based on FSC/SSC and the frequencies of expression for NK cell marker CD16 + CD56+ PE (y axis) and CD3 (x axis) were determine for each time point. Table S1. Evaluation of RNA in PBMCs samples. The quantity and quality of the extracted RNA was evaluated in the Nanodrop ND-1000 spectrophotometer taking into account the ratio of absorbance 260/280 and 260/230. Figure S2. Evaluation of RNA integrity in PBMCs samples. Agarose gel electrophoresis of RNA samples for confirmation of RNA integrity by inspection of the 28S and 18S rRNA bands. Control samples: Lanes 1, 2, 3, 10, 14, 18, 22 and 26; 7 h samples: Lanes 4, 5, 6, 11, 15, 19, 23 and 27; 12 h samples: Lanes 12, 16, 20, 24, and 28; 24 h samples: Lanes 7, 8, 9, 13, 17, 21, 25 and 29. Figure S3. Evaluation of GAPDH gene expression in PBMCs. Gene expression was measured by qRT-PCR, each figure represents the CT value and its stability over time for samples from each volunteer. Individual and mean values are shown. No statistical differences were observed as estimated by one-way ANOVA followed by Dunnett’s multiple comparison test. (DOCX 446 kb) [file 12865_2019_286_MOESM1_ESM.docx]

**Additional file 1: Figure S1. Representative dot plot showing the effect of sample processing time in CD3+ expression in lymphocyte population from whole blood.** Lymphocyte population was gated based on FSC/SSC and the frequencies of expression for NK cell marker CD16+CD56+ PE (y axis) and CD3 (x axis) were determine for each time point.


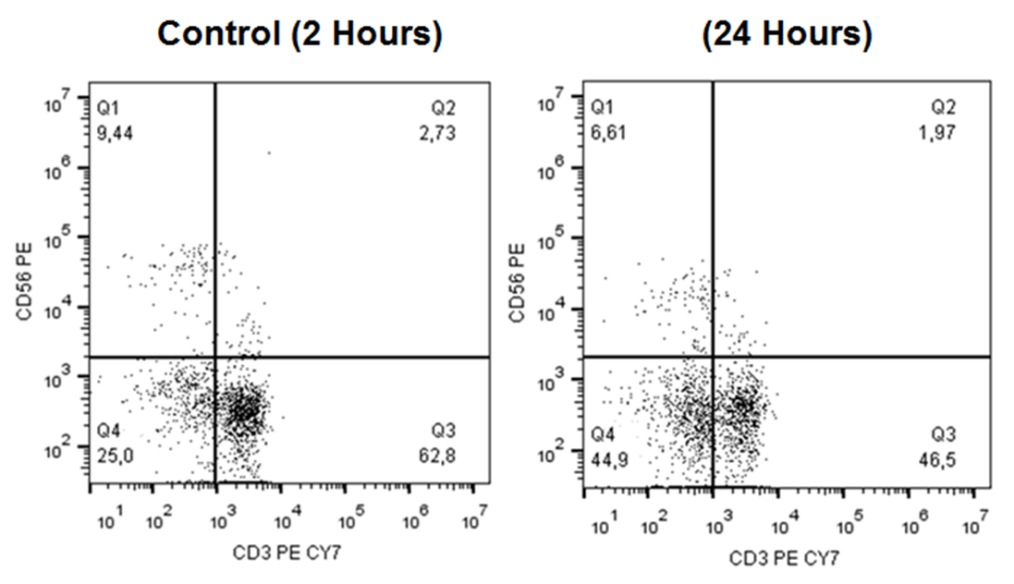


**Additional file 1: Table S1. Evaluation of RNA in PBMCs samples.** The quantity and quality of the extracted RNA was evaluated in the Nanodrop ND-1000 spectrophotometer taking into account the ratio of the absorbance 260/280 and 260/230.

| **Well** | **Sample ID** | **ng/µl** | **260/280** | **260/230** | **Well** | **Sample ID** | **ng/µl** | **260/280** | **260/230** |
| --- | --- | --- | --- | --- | --- | --- | --- | --- | --- |
|  |  |  |  |  |  |  |  |  |  |
| **1** | **WT1001 2H** | 298,27 | 1,93 | 1,31 | **16** | **WT1010 12H** | 621,26 | 1,93 | 1,64 |
| **2** | **WT1004 2H** | 366,41 | 1,92 | 1,28 | **17** | **WT1010 24H** | 582,02 | 1,97 | 1,53 |
| **3** | **WT1005 2H** | 230,89 | 1,89 | 1,29 | **18** | **WT1011 2H** | 627,47 | 1,96 | 1,67 |
| **4** | **WT1001 7H** | 250,18 | 1,88 | 1,07 | **19** | **WT1011 7H** | 572,45 | 2 | 1,64 |
| **5** | **WT1004 7H** | 257,89 | 1,89 | 1,28 | **20** | **WT1011 12H** | 408,06 | 1,92 | 1,43 |
| **6** | **WT1005 7H** | 393,25 | 1,9 | 1,54 | **21** | **WT1011 24H** | 699,37 | 2,01 | 1,78 |
| **7** | **WT1001 24H** | 310,28 | 1,92 | 1,44 | **22** | **WT1012 2H** | 534,33 | 1,95 | 1,94 |
| **8** | **WT1004 24H** | 355,4 | 1,91 | 1,47 | **23** | **WT1012 7H** | 433,01 | 1,91 | 1,91 |
| **9** | **WT1005 24H** | 282,38 | 1,9 | 1,14 | **24** | **WT1012 12H** | 398,31 | 1,92 | 1,73 |
| **10** | **WT1009 2H** | 752,62 | 1,95 | 1,78 | **25** | **WT1012 24H** | 379,41 | 1,92 | 1,8 |
| **11** | **WT1009 7H** | 654,77 | 1,98 | 1,32 | **26** | **WT1013 2H** | 380,96 | 1,93 | 1,59 |
| **12** | **WT1009 12H** | 519,82 | 2,02 | 1,41 | **27** | **WT1013 7H** | 616,23 | 1,95 | 2,08 |
| **13** | **WT1009 24H** | 655,22 | 1,98 | 1,92 | **28** | **WT1013 12H** | 406,7 | 1,91 | 1,53 |
| **14** | **WT1010 2H** | 694,04 | 1,95 | 1,98 | **29** | **WT1013 24H** | 539,66 | 1,92 | 1,8 |
| **15** | **WT1010 7H** | 559,05 | 1,99 | 1,99 |  |  |  |  |  |

**Additional file 1: Figure S2. Evaluation of RNA integrity in PBMCs samples.** Agarose gel electrophoresis of RNA samples for confirmation of RNA integrity by inspection of the 28S and 18S rRNA bands was performed. **Control samples: Lanes** 1, 2, 3, 10, 14, 18, 22 and 26; **7h samples: Lanes** 4, 5, 6, 11, 15, 19, 23 and 27; **12h samples: Lanes 12, 16, 20, 24, and 28; 24h samples: Lanes** 7, 8, 9, 13, 17, 21, 25 and 29**.**

**Additional file 1: Figure S3. Evaluation of GAPDH gene expression in PBMCs obtained from whole blood.** Gene expression was measured by qRT-PCR, each figure represents the CT value and its stability over time for samples from each volunteer. Individual and mean values are shown. No statistical differences were observed as estimated by one-way ANOVA followed by Dunnett´s multiple comparison test.
